# Spinal cord ischemia following open surgery of a ruptured isolated internal iliac artery aneurysm: A case report

**DOI:** 10.1097/MD.0000000000027619

**Published:** 2021-10-29

**Authors:** Kentaro Akabane, Tetsuro Uchida, Rieko Umetsu, Shuto Hirooka, Cholus Kim, Hideaki Uchino, Takao Shimanuki

**Affiliations:** a Division of Cardiovascular Surgery, Nihonkai General Hospital, Sakata, Japan; b Second Department of Surgery, Yamagata University Faculty of Medicine, Yamagata, Japan.

**Keywords:** isolated internal iliac artery aneurysm, paraplegia, spinal cord ischemia

## Abstract

**Introduction:**

Isolated internal iliac artery (IIA) aneurysms (IIIAAs) rarely occur. However, they may enlarge asymptomatically and rupture, causing fatality. Even after successful surgery of ruptured IIIAAs, there might be a potential risk of postoperative spinal cord ischemia (SCI)-related paraplegia, which is extremely rare. However, this paraplegia significantly impacts patients’ activities of daily living.

**Patient concerns:**

A 71-year-old man who had no remarkable medical history was referred to our hospital with sudden lower abdominal pain.

**Diagnosis:**

Computed tomography (CT) revealed right IIIAA with small volumes of contrast medium extravasation and hematoma. He presented with cyanosis in the bilateral lower limbs. Moreover, blood gas analysis showed lactic acidosis. Therefore, he was diagnosed with ruptured IIIAA complicated by peripheral circulatory failure.

**Interventions:**

Considering his pre-shock status, an emergency operation comprising ligation of the proximal neck and suture closure of the distal IIA orifice was successfully performed.

**Outcomes:**

Immediately after surgery, motor and sensory dysfunction in the bilateral lower limbs occurred. Magnetic resonance imaging confirmed the presence of SCI. The patient could not stand independently and had neurogenic bladder and rectal disorder.

**Conclusion:**

Postoperative SCI is a serious complication with no definitive predictors, preventive methods, or highly efficacious treatments. Therefore, vascular surgeons should preempt its occurrence and focus on preventing hemodynamic instability and maintain collateral extra-segmental arterial blood flow, especially in ruptured cases.

## 1 Introduction

Isolated internal iliac artery (IIA) aneurysms (IIIAAs) rarely occur.^[1,2]^ However, they may enlarge asymptomatically, rupture, and cause fatality.^[3]^ Additionally, even after successful treatment of ruptured IIIAAs, a potential risk of postoperative spinal cord ischemia (SCI)-related paraplegia remains, which is a rare condition. This paraplegia significantly disrupts patients’ abilities to perform activities of daily living. Herein, we report a case of postoperative paraplegia due to SCI in a patient who underwent open surgical repair of ruptured IIIAA on an emergency basis.

## 2 Case presentation

**Figure 1:**
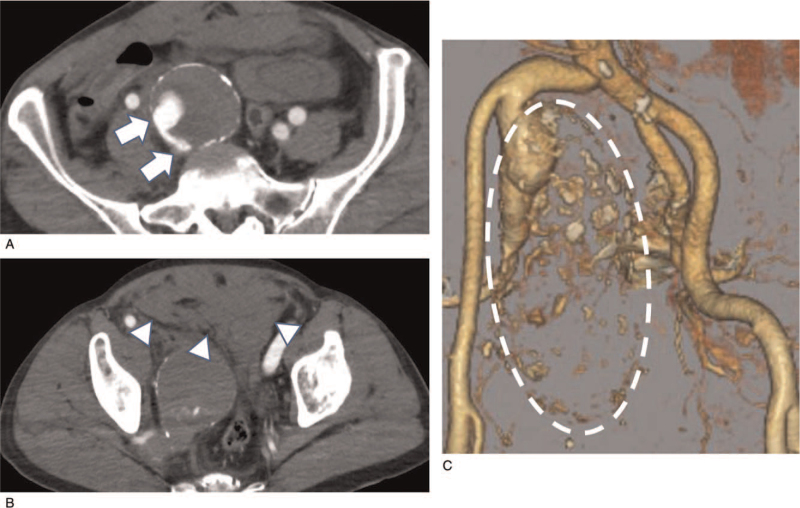
Preoperative CT. (A) CT reveals a giant IIIAA with a dimeter of 67 × 74 mm. Thick mural thrombus, and small volumes of contrast medium extravasation into the aneurysmal wall are observed (arrow). (B) Hematomas are found in the intraperitoneal and pelvic spaces (arrow head). (C) Three-dimensional CT shows a giant IIIAA (dotted line). CT = computed tomography, IIIAA = isolated internal iliac artery aneurysm.

A 71-year-old man with no remarkable medical history experienced sudden lower abdominal pain. Computed tomography (CT) performed at a neighboring hospital revealed a right IIIAA (6.7 cm in diameter) with small volumes of contrast medium extravasation and hematoma (Fig. 1). He was referred to our hospital due to a suspected ruptured right IIIAA. On emergency room arrival, the patient's general physical condition was stable (blood pressure, 120/70 mmHg; heart rate, 120 bpm). He complained of lower abdominal tenderness upon palpation and had bilateral lower limb cyanosis. Blood gas analysis showed lactic acidosis (pH, 7.258; lactate, 6.5 mmol/L). He was diagnosed with a ruptured right IIIAA complicated by peripheral circulatory failure. Considering his pre-shock status and probable time-consuming preparation for endovascular devices, emergency open surgical aneurysm repair was scheduled.

**Figure 2:**
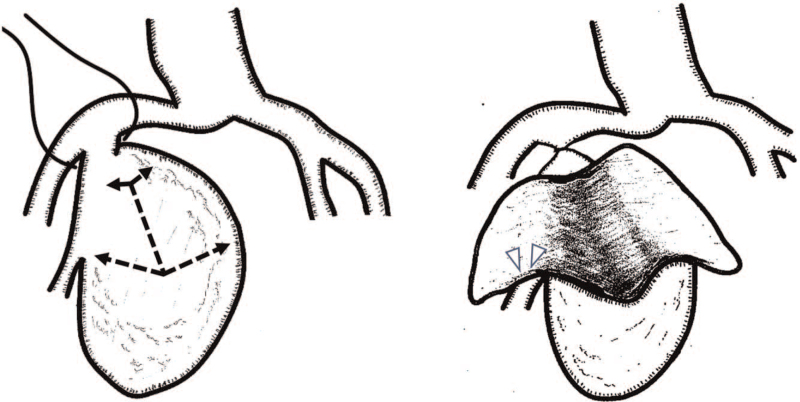
Schematic drawings of surgery. (A) The proximal neck of IIA is encircled and ligated, and the aneurysm is opened (dotted lines). (B) After large amounts of mural thrombi are removed, ostium of distal IIA is closed from the inside of the aneurysm (arrow head). IIA = internal iliac artery.

Under general anesthesia, the abdominal cavity was entered through a lower midline skin incision. The intraperitoneal and retroperitoneal cavities were filled with large volumes of hematomas. After surgical hematoma evacuation, the large right IIIAA was easily identified in the pelvic cavity. The proximal neck of the right IIIAA was encircled carefully and ligated close to the right common iliac artery. Active bleeding from the aneurysmal surface was not observed. Encircling the distal IIA was considered difficult; therefore, we opened the aneurysm and removed a large mural thrombus and performed suture closure of distal IIA drainage sites (Fig. 2). Circulatory status was stable throughout the operation. Perioperative hypotension was not observed.

**Figure 3:**
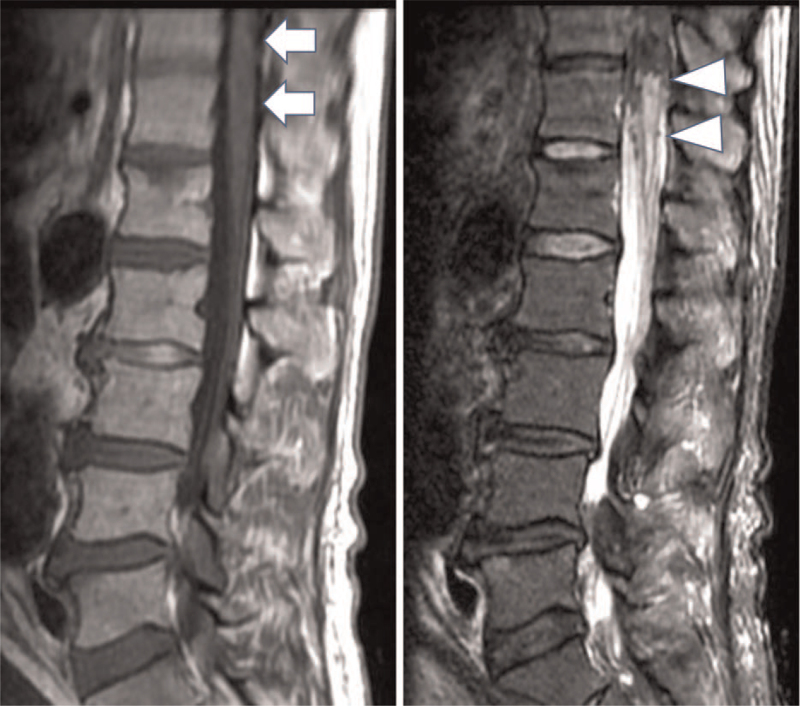
Spinal cord infarction is identified on MRI. (A) T1WI (arrow). (B) T2WI (arrow head). MRI = magnetic resonance imaging, WI = weighted image.

The patient was weaned from the ventilator immediately after the operation. However, he was found to have motor dysfunction from the bilateral thighs to the toes (Manual Muscle Testing of 1/1) and sensory disturbance below the L5 region. Spinal magnetic resonance imaging (MRI) confirmed presence of SCI at T12-L1 (Fig. 3). The patient was treated with cerebrospinal fluid drainage and continuous intravenous infusion of naloxone. Although SCI was intensively managed, he could not stand independently, and neurogenic bladder and rectal disorder remained. Thereafter, after 51 postoperative days, he was transferred to the rehabilitation center.

## 3 Discussion

Isolated aneurysm of the IIA without abdominal aortic aneurysms (AAA) is rare with reported incidence rates of 0.9% to 1.9%.^[1]^ Among these, isolated common iliac artery aneurysm is the most common. However, the incidence of IIIAA is even lower, that is, 0.4%.^[2]^ Generally, IIIAAs lie deep in the pelvis and cannot be palpated from the body surface. Therefore, IIIAAs often remain asymptomatic and are unrecognized until they rupture. In many cases of IIIAAs, rupture may be first presented with a shock status. In fact, approximately 40% of IIIAAs are detected as ruptured IIIAA with associated mortality rates >50%^[3]^.

There is no consensus on surgical indication for non-ruptured IIIAAs regarding its diameter. In patients with IIIAA exceeding 3 cm in diameter, the risk of rupture has been reported to be 14% to 31%, and surgical treatment should be considered.^[2]^ However, regarding IIIAAs with diameter <3 cm, investigators deemed them to have the potential to rupture, as the risk of rupture not necessarily correlated with the aneurysmal diameter.^[3]^

Regarding open surgical repair of ruptured IIIAAs, resection of aneurysm and vascular reconstruction with prosthetic graft might be ideal. However, there are technical difficulties of reconstruction of atherosclerotic distal IIA because it lies deep in the pelvis, as experienced in our case. If the proximal neck is long enough to be encircled, ligation of the proximal neck and suture closure of the distal IIA orifice can be performed in most cases. As the endovascular era evolves, stent-graft placement into the common iliac artery and coil embolization of the distal IIA has been considered as an effective alternative to conventional open surgery.^[2]^ In our case, although hemodynamic instability was not observed, considering peripheral cyanosis suggestive of a pre-shock status and the time required for preparing endovascular devices, open surgery was performed immediately.

Paraplegia owing to SCI is a serious postoperative complication of descending thoracic aortic and thoracoabdominal aortic aneurysms surgeries.^[4]^ To avoid SCI, adequate spinal cord blood supply should be ensured throughout surgery. The largest of segmental arteries known as the “Adamkiewicz artery," branched from the T9 to T12 level of the aorta in 3 of 4 cases.^[5]^ Therefore, regarding infrarenal aortic and iliac aneurysm surgery, this important segmental artery should not be sacrificed. SCI is a rare complication (incidence rate of 0.1%–0.2% in unruptured cases and 2.0% in ruptured cases). However, SCI possibly occurred in patients who underwent abdominal aortic surgical repair.^[6]^ Griepp et al reported the importance of extra-segmental arteries for inflow into the spinal cord, in a so-called “collateral network concept.”^[7]^ They also indicated that steal from spinal-cord nutrient blood flow through an alternate low resistance pathway was related to the pathogenesis of SCI. The IIA branches into the iliolumbar and external sacral arteries, which are crucial for maintaining collateral blood flow into the lower spinal cord.^[8,9]^ When unilateral IIA blood flow is impaired by surgery, SCI may not occur due to collateral blood flow from the contralateral IIA. Conversely, in cases of emergency surgery for ruptured AAA and IIIAA, spinal cord blood flow through the rupture site could be disrupted. This induces decreased spinal cord collateral flow, low blood pressure, and subsequent SCI. Impaired blood flow through collateral network has a multifactorial pathogenesis and is deemed to be associated with postoperative SCI in this particular setting.

Cerebrospinal fluid drainage and continuous intravenous naloxone administration are deemed effective in SCI. However, they are not expected to induce absolute amelioration. Moreover, 50% of cases with paraplegia show no neurological recovery.^[10]^ Paraplegia due to SCI is a serious complication. There are no definitive predictors, preventive methods, or effective treatments for it. Hence, vascular surgeons should preempt SCI-induced paraplegia and focus on prevention of hemodynamic instability and maintain collateral blood flow network by extra-segmental arteries, especially in ruptured cases.

## Author contributions

**Conceptualization:** Kentaro Akabane, Rieko Umetsu, Shuto Hirooka, Cholus Kim, Hideaki Uchino, Takao Shimanuki.

**Supervision:** Takao Shimanuki.

**Writing – original draft:** Kentaro Akabane.

**Writing – review & editing:** Tetsuro Uchida.
